# Electrochemical Recovery of Rare-Earth Elements from Coal Fly Ash Using Ionic Liquids as both Extractant and Electrolyte

**DOI:** 10.1021/acs.est.5c16688

**Published:** 2026-05-27

**Authors:** Anuja Tripathi, Ting Liu, Joe F. Bozeman, Ching-Hua Huang, Xing Xie

**Affiliations:** † School of Civil and Environmental Engineering, 1372 Georgia Institute of Technology, 311 Ferst Drive NW, Atlanta, Georgia 30332, United States; ‡ Jimmy and Rosalynn School of Public Policy, 1372 Georgia Institute of Technology, 258 fourth St NW, Atlanta, Georgia 30332, United States

**Keywords:** ionic liquids, rare-earth elements recovery, coal fly ash

## Abstract

Rare-earth elements (REEs) are critical for medical technologies, electronics, and clean energy. Coal fly ash (CFA), a byproduct of coal combustion, offers a promising alternative REE source. However, efficient extraction and separation of REEs from CFA remain challenging due to the complex composition of CFA. This study introduces a sustainable method for REE recovery using a recyclable ionic liquid, betainium bis­(trifluoromethylsulfonyl)­imide ([Hbet]­[Tf_2_N]), which serves both as the extractant from CFA and as the electrolyte in electrodeposition. In the first stage, [Hbet]­[Tf_2_N] preferentially extracts REEs from CFA through leaching. In the second stage, the REE-enriched ionic liquid undergoes electrochemical deposition using amperometry techniques, where REEs are reduced and deposited onto the electrode. The deposition experiments were conducted from −0.5 to −2.0 V vs a Pt quasi-reference electrode in a three-electrode setup comprising titanium as the working electrode and platinum as both the reference and counter electrodes. Varying the applied potential enabled potential-dependent preferential REE deposition. At −0.5 V, neodymium (Nd) showed preferential recovery, reaching 25% with a separation factor of 37 over other REEs. In contrast, applying a more negative potential increased overall deposition, yielding ∼50% Nd recovery and 10–20% recovery for the remaining REEs. After recovery, the ionic liquid was regenerated and reused for a subsequent electrochemical recovery cycle. Overall, this study demonstrates a feasible approach for REE recovery from CFA waste, with potential to enhance resource utilization within the REE supply chain.

## Graphical abstract

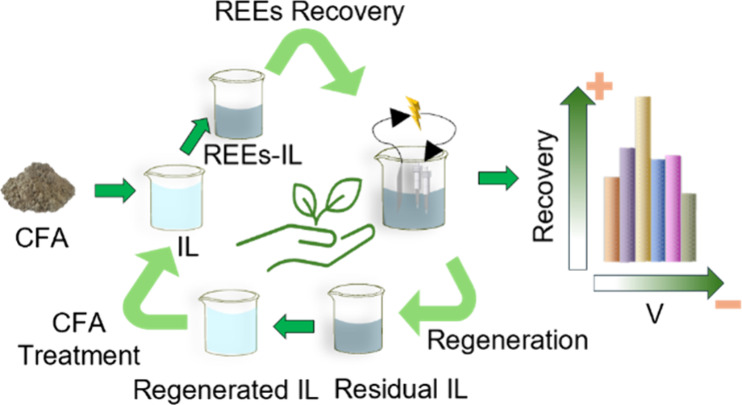

## Introduction

Rare-earth elements (REEs), including the 15 lanthanide elements, are vital to modern technologies because of their distinctive physicochemical characteristics and essential functions in clean energy, defense, and various industrial applications such as catalysis, batteries, semiconductors, and magnetic materials.^1–4^ However, conventional REE mining causes deforestation, CO_2_ emissions, and radioactive and heavy-metal pollution that damage ecosystems.^5,6^ Coal fly ash (CFA), a byproduct of coal combustion, offers a promising alternative REE source owing to its enriched REE content and vast reserves accumulated in disposal sites and industrial stockpiles. In the United States alone, over 40 million tons of CFA are generated annually.^7–9^ Although coal contains REEs only in trace amounts, combustion concentrates these elements in the ash, making CFA a comparatively enriched secondary resource for REEs.^10^ Yet, less than 40% of CFA is currently reused, mainly in concrete production, while the rest is landfilled or impounded.^11^ Recovering REEs from CFA could offer a dual benefit by alleviating supply shortages of REEs while mitigating the environmental and economic impacts associated with CFA disposal.^8^

Previous studies on REE recovery from CFA have largely relied on harsh conditions involving concentrated acids, high temperatures, or corrosive reagents.^8,12^ For instance, Taggart et al. achieved >70% leaching by sintering CFA with NaOH at 450 °C followed by 2 M HNO_3_ leaching,^13^ whereas other approaches employed 15 M HNO_3_ at 90 °C, making the process highly energy- and chemical-intensive.^8,14^ Milder processing conditions typically result in a substantial loss of extraction efficiency. Deng et al. combined electrothermal preactivation with diluted HCl, in which leaching was improved but REE selectivity was poor.^15^ In addition, various methods, including acid leaching, solvent stripping, and hydrometallurgical techniques, have been investigated for REE recovery from CFA.^16–20^ Although acid digestion can extract REEs, it also dissolves heavy metals and requires large reagent volumes.^21^ Sorption and membrane-based techniques offer limited selectivity due to cation interference and produce secondary waste from repeated acid and solvent use.^22,23^ Overall, these approaches remain corrosive, energy-intensive, and environmentally unsustainable, and large-scale industrial recovery has yet to be achieved. An ideal process would enable high REE extraction efficiency while minimizing chemical and water use, reducing waste generation, and limiting interference from coexisting elements.^24^

In contrast to conventional methods, our recent work showed that the ionic liquid (IL) betainium bis­(trifluoromethylsulfonyl)­imide ([Hbet]­[Tf_2_N]) offers a greener alternative owing to its favorable properties.^25–27^ Our previous work has demonstrated that REEs can be preferentially extracted from CFA using the [Hbet]­[Tf_2_N], along with minor amounts of coextracted elements.^25,26,28^ The [Hbet]­[Tf_2_N]-based extraction facilitates metal leaching and separation through thermally induced phase transitions with water, forming a single homogeneous phase at elevated temperatures and separating upon cooling to concentrate REEs in the IL phase. The REEs-enriched IL phase can then be stripped to remove the REEs, allowing the IL to be regenerated and reused. Although [Hbet]­[Tf_2_N] can effectively extract REEs from CFA, achieving selective separation among individual REEs remains one of the most critical and technically demanding challenges due to REEs’ similar chemical properties. Meanwhile, electrochemical recovery has emerged as a promising approach, demonstrating effective REEs recovery for elements such as Y and Eu from lamp phosphors, Nd from NdFeB magnets, and several other REEs from synthetic systems.^29–33^ Despite being promising, prior studies on electrochemical REEs recovery have depended on acid digestion and inert-atmosphere conditions, limiting scalability and selectivity while requiring hazardous reagents and substantial water use. Ionic liquids such as [Hbet]­[Tf_2_N] exhibit multiple favorable properties, including high conductivity, a wide electrochemical window, recyclability, low flammability, and negligible vapor pressure, suggesting viability as a sustainable and recyclable electrolyte for electrochemical recovery of REEs.^33^ Since REE^3+^ reduction requires highly negative potentials and nonaqueous environments to prevent hydrolysis, the nonvolatile, hydrogen-bonding characteristics of [Hbet]­[Tf_2_N] provide a stable medium that can enable controlled and potential-dependent preferential REE^3+^ reduction. To date, however, no studies have demonstrated the electrochemical recovery of REEs from [Hbet]­[Tf_2_N] following leaching of real CFA samples, and existing reports using this IL have not achieved potential-dependent preferential separation among individual REEs. Demonstrating this feasibility would mark a significant step toward scalable, environmentally sustainable electrochemical REE recovery with potential-dependent preferential deposition.

In this study, we present a novel and integrated approach for the potential-dependent preferential deposition of REEs from CFA using the ionic liquid [Hbet]­[Tf_2_N] as both the extractant and the electrolyte. As shown in Figures 1a,b and S1, a Class C CFA generated from coal combustion was first treated with [Hbet]­[Tf_2_N] to leach REEs. The [Hbet]­[Tf_2_N] IL exhibits thermomorphic behavior, where controlled water content enables temperature-dependent phase separation and enhances the dissolution of rare-earth oxides through partial hydration and improved solvation of REE species. After this separation, REEs bind preferentially to the IL phase compared to other metals, yielding a REE-enriched IL, which is subsequently used for electrochemical recovery in a three-electrode system under a constant potential. To our knowledge, this is the first demonstration of such dual functionality—combining extraction and electrochemical recovery—of [Hbet]­[Tf_2_N] for REE separation from real CFA samples. The broad electrochemical stability of this IL enables REE reduction at potentials inaccessible in aqueous systems, allowing potential-dependent preferential REE deposition while minimizing corecovery of heavy metals, salts, and impurities. Moreover, this process operates under ambient conditions, offering high selectivity, reusability, minimal waste generation, and enhanced sustainability for scalable REE recovery.

**Figure 1:**
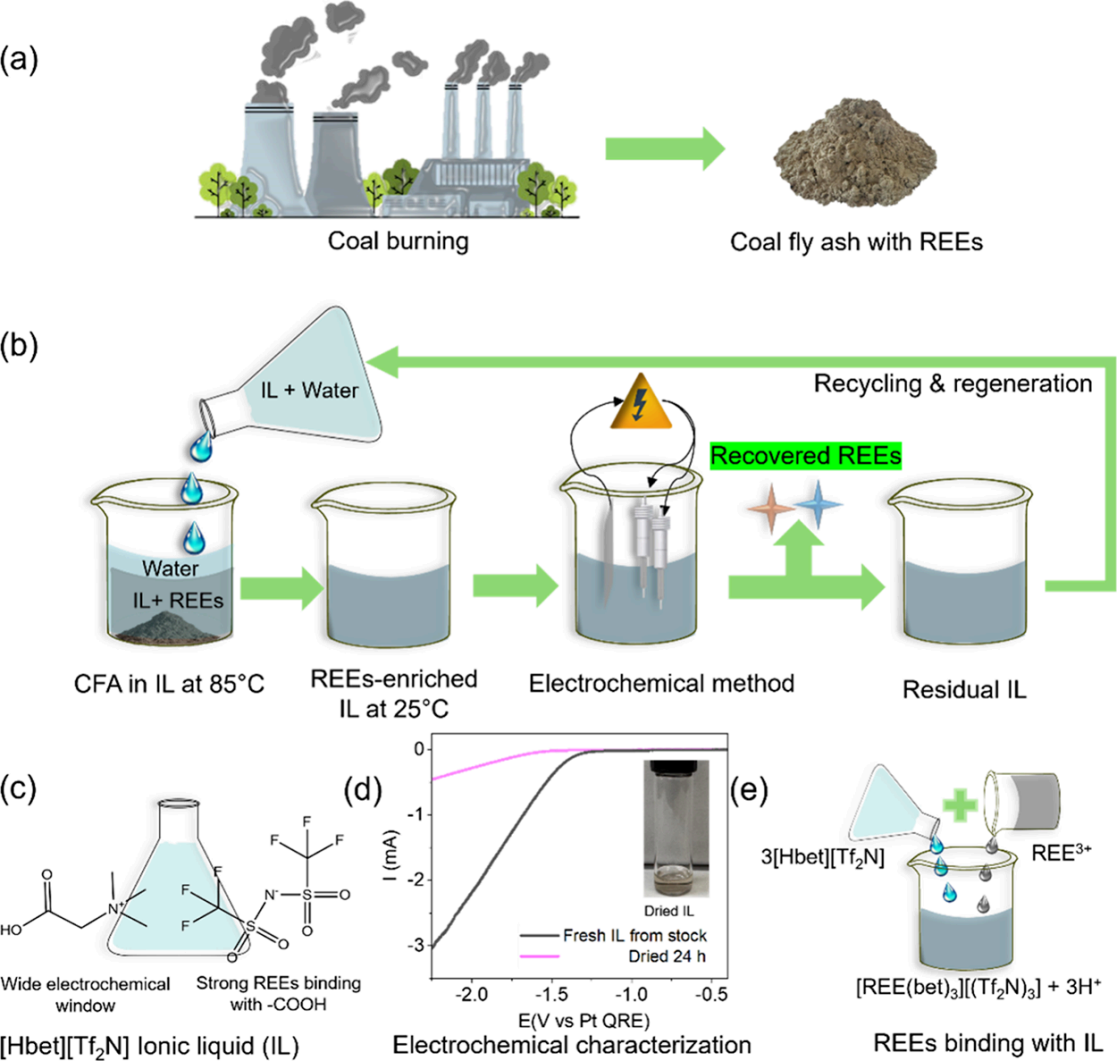
Addressing challenges for sustainable REE recovery: (a) CFA generated from coal combustion is pretreated to remove impurities and salts; (b) the purified CFA undergoes ionic liquid (IL) extraction using [Hbet]­[Tf_2_N], followed by electrochemical deposition of REEs in a three-electrode cell. The IL is then regenerated and recycled for reuse; (c) chemical structure of [Hbet]­[Tf_2_N] and its electrochemical stability window comparing fresh and dried IL; (d) electrochemical characterization of the ionic liquid and dried ionic liquid using linear sweep voltammetry; and (e) schematic illustration of REE–IL complexation during extraction.

## Results

### Extraction of REEs from CFA with [Hbet]­[Tf_2_N]

The IL-based extraction and separation of REEs from 93927 CFA (fly ash derived from a power plant burning Class C Powder River Basin coals) solids was adapted from our previously reported study.^28^ In brief, the [Hbet]­[Tf_2_N]-based process enables single-step REE recovery from CFA solids by utilizing its thermomorphic behavior, allowing concurrent REE leaching and enrichment into the IL phase upon heating at 85 °C and cooling (Figures 1b and S1). The REE extraction in the IL occurs through a proton-exchange mechanism (Figure 1e), in which three protons from the IL’s carboxylic group are exchanged with one REE^3+^ ion, forming stable REE–IL complexes. After 3 h of leaching at 85 °C, betaine monohydrate and ascorbic acid are introduced to promote REE transfer and suppress heavy-metal coextraction. Upon cooling, the IL phase becomes enriched with REEs, while bulk and trace elements largely remain in the aqueous layer. The resulting extraction data show 48.5% total REEs extracted from CFA along with additional elements such as Al, Fe, Ni, and Se (Table S1 and Figure S2).

### Electrochemical Characterization of [Hbet]­[Tf_2_N]

The electrochemical stability window of [Hbet]­[Tf_2_N] was examined using linear sweep voltammetry (LSV) for both the as-received and 24 h-dried samples (Figure 1d). All potentials are reported relative to the Pt quasi-reference electrode (QRE), which was calibrated against the ferrocene/ferrocenium (Fc/Fc^+^) redox couple at −0.02 V (Figure S3). The LSV results indicate a wide electrochemical window, showing the IL’s stability and suitability for nonaqueous electrochemical applications. The as-received [Hbet]­[Tf_2_N] IL exhibited a reduction window (−1.4 to −2.5 V vs Pt QRE) compared to the dried [Hbet]­[Tf_2_N] (−1.5 to −2.5 V vs Pt QRE), which might be due to residual water presence in the purchased IL. The water molecules likely led to electrolysis, reducing the electrochemical stability relative to the dried IL. A similar trend has been reported for hydrophobic ionic liquids, where increasing water content narrows the electrochemical stability window.^33,34^ Consequently, the REE-enriched IL obtained after extraction was vacuum-dried at 120 °C for 24 h prior to electrochemical experiments. As shown in Figure 2a, REE-enriched IL extracted from CFA displayed distinct reduction peaks at −0.38 V vs Pt QRE and −1.17 V vs Pt QRE. However, REEs typically exhibit reduction potentials in the low negative range of −1.7 to −2.9 V (Table S2). Notably, Fe and Ni, present in the ash, were coextracted into the IL at concentrations of 393 and 0.3 mg/L, respectively (Table S1 and Figure S2). The shift toward more positive potentials is likely attributed to the presence of coextracted Fe and Ni, whose lower reduction potentials facilitate synergistic coreduction, thereby reducing the electron-transfer barrier and shifting the apparent reduction potential.^35–37^

**Figure 2:**
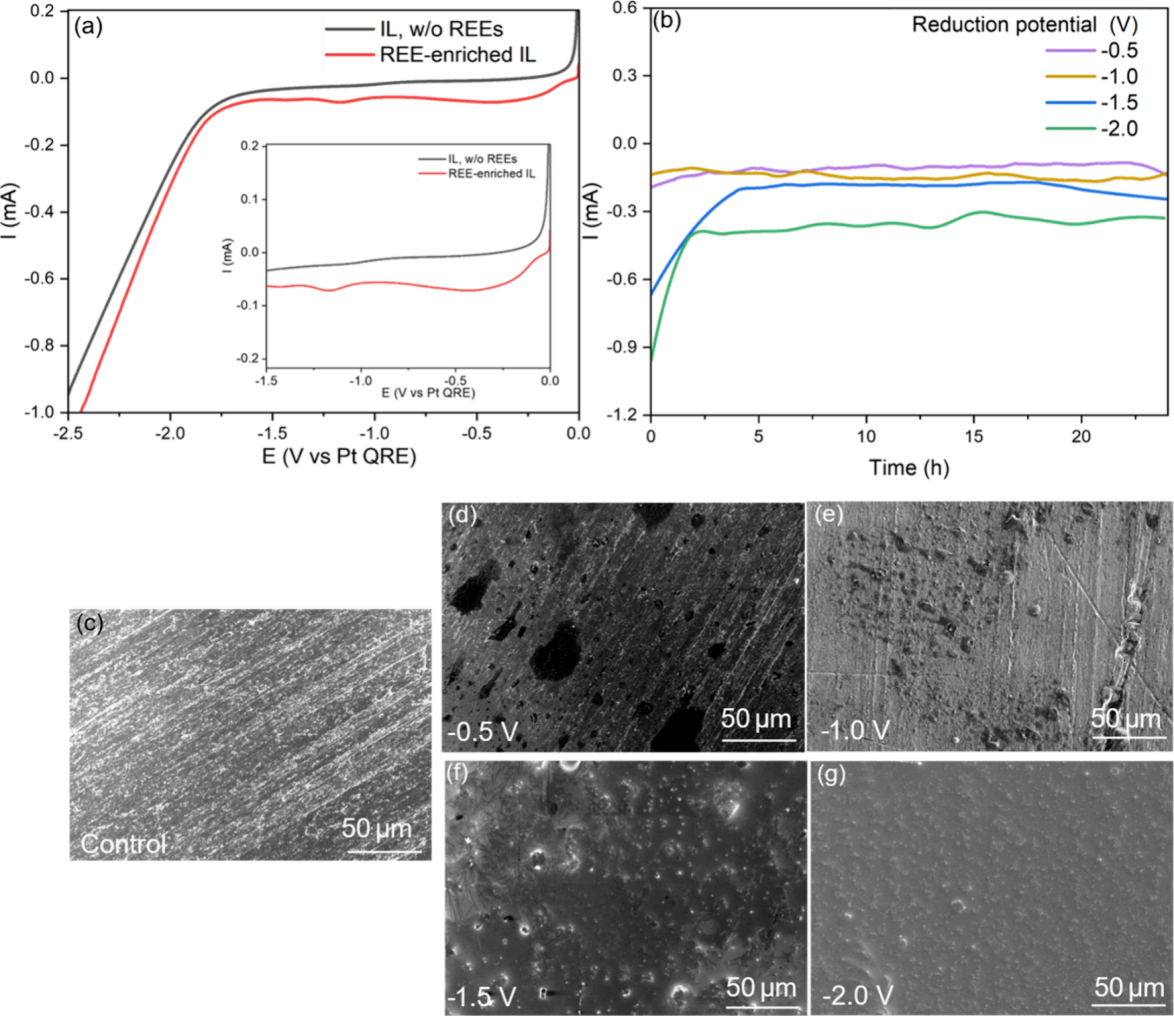
(a) LSV on the [Hbet]­[Tf_2_N] ionic liquid (black curve) and REE-enriched IL from CFA extraction (red curve) in a three-electrode electrochemical setup. Ti foil as the working electrode and Pt as the reference and counter electrode. Inset image is the zoomed-in figure between 0 V vs Pt QRE and −2.0 V vs Pt QRE. (b) Chronoamperometry technique in REEs-enriched IL for 24 h over the potential range of −0.5 V vs Pt QRE to −2.0 V vs Pt QRE. (c–g) Scanning electron microscopy images of control Ti surface (c), at constant potentials of −0.5 (d), −1.0 (e), −1.5 (f), and −2.0 V (g) for 24 h. QRE: quasi-reference electrode.

### Electrochemical Recovery of REEs

To recover REEs from the REE-enriched IL, chronoamperometry (CA) was performed at −2.0 V versus Pt QRE, −1.5 V versus Pt QRE, −1.0 V versus Pt QRE, and −0.5 V versus Pt QRE (Figure 2b). The −2.0 V vs Pt QRE was selected based on the typical reduction potentials of REEs (Table S2), and a more negative potential could lead to degradation of the IL. Chen et al. showed that at highly negative potentials, the cation of the ionic liquid can undergo reduction at the electrode surface, leading to ionic liquid breakdown and limiting its electrochemical stability window.^38^ The −1.5 V vs Pt QRE, −1.0 V vs Pt QRE, and −0.5 V vs Pt QRE were chosen as they remain within the IL’s electrochemical stability window. After CA deposition for 24 h at low negative potentials, a trace appearance of electrodeposited elements was visibly observed on the Ti foil as the working electrode (Figure S4). The morphological characterization of the Ti foil after CA was performed using scanning electron microscopy (SEM) and atomic force microscopy (AFM) (Figures 2c–g and S5). The untreated Ti foil showed a smooth surface without noticeable features. In contrast, the Ti foil electrochemically treated at −0.5 V vs Pt QRE displayed partial surface coverage compared to the control, which became increasingly pronounced at more negative potentials, indicating possible IL deposition or irregular metal co-deposition. AFM analysis further revealed a monotonic increase in surface roughness with increasing negative potential, rising from 15.1 ± 1.49 nm at −0.5 V to 19.13 ± 2.05 nm at −1.0 V, 23.5 ± 1.95 nm at −1.5 V, and 28.26 ± 3.18 nm at −2.0 V, compared to 13.8 ± 0.5 nm for pristine Ti foil. The enhanced surface roughness at higher negative potentials further supports the occurrence of deposition. At −2.0 V, the surface appeared fully covered by the IL, likely representing a combination of IL, REEs, and co-extracted metals deposition, as shown by the elemental characterization in the next section.

To investigate elemental characterization on Ti electrodes after electrochemical deposition at −0.5 V vs Pt QRE, −1.0 V vs Pt QRE, −1.5 V vs Pt QRE, and −2.0 V vs Pt QRE for 24 h, X-ray photoelectron spectroscopy (XPS) was performed (Figure 3). The untreated Ti control showed no detectable REE peaks, whereas the electrochemically deposited samples revealed the presence of REEs. The Y signals at −1.5 V vs Pt QRE and −2.0 V vs Pt QRE indicated successful electrodeposition from the REE-enriched IL. La and Ce peaks were also observed at −1.0 V vs Pt QRE, −1.5 V vs Pt QRE, and −2.0 V vs Pt QRE, consistent with their progressive reduction and surface incorporation at higher cathodic potentials.^39,40^ Gd peaks at ∼144 eV and Dy 3d peaks at 1305 eV were both detected only at −2.0 V, indicating their deposition occurred at this potential, consistent with their standard reduction potential.^41^ At −0.5 V, Nd was the sole REE detected, and its presence remained as the potential was further decreased to −2.0 V. The characteristic peaks of Nd in its metallic and oxide forms appeared at 978, 980.6, and 981 eV, confirming its electrodeposition from the REE-enriched IL.^42^ Nd reduction at −0.5 V occurs at a more positive potential than its standard value, likely because Fe reduces near −0.5 V (Tables S1 and S2) and strongly binds with Nd to form Fe–Nd alloys that promote Nd reduction. Similar co-deposition phenomena have been previously demonstrated, in which REE reduction often may lead to the formation of metallic or metal–oxide phases depending on the electrolyte composition and ambient conditions. For instance, Xu et al. achieved electrodeposition of rare-earth–iron alloys from ionic liquid electrolytes, identifying both metallic and oxidized REE species depending on potential and moisture.^43^ They showed that Nd–Fe co-deposition arises from a surface-mediated process in which REE and transition-metal (TM) ions first adsorb onto the cathode. The TM (e.g., Fe) is then preferentially reduced to form reactive metallic sites that facilitate interfacial energy transfer and transient TM–TM and TM–REE complexes, thereby lowering the effective reduction barrier and enabling REE incorporation into the deposit. This might be due to continuous surface-mediated interactions that allow reduction and alloying to proceed simultaneously, potentially facilitating TM–REE co-deposition.^43^ Consistent with this mechanism, our synthetic electrolyte studies further showed that Nd recovery was significantly enhanced in the presence of Fe compared to Nd alone in the IL system (Figure S6). Likewise, Cvetković et al. investigated Nd deposition from NdF_3_–LiF–Nd_2_O_3_ melts on Mo electrodes and identified both metallic Nd^0^ and Nd_2_O_3_ phases, confirming that surface oxidation of reduced REEs readily occurs during or after deposition.^44^ Although XPS can distinguish oxidation states via binding-energy shifts, the observed signals mainly reflect surface oxidation rather than the underlying metallic layer, suggesting that metallic REEs were initially deposited but partially oxidized during air exposure.^45^ Because coal fly ash contains multiple transition metals, other TMs may similarly participate in surface-mediated pathways analogous to the hypothesized Nd–Fe co-deposition mechanism. To further identify the co-deposited elements, XPS was also performed on elements at similar reduction potentials, such as S, Ni, Fe, Se, Al, and F (Figure S7). None of these elements were detected in the untreated Ti control. Fe and Ni, in metallic or oxide forms, were detected at all potentials, consistent with their reduction potential range that enables their reduction at more positive potentials than the REEs.^46–49^ Se showed a characteristic peak at 55.2 eV at −1.5 V, while Al was detected in both metallic and oxide forms at −1.5 and −2.0 V. F and SO_4_^2–^ species were consistently present across all potentials, originating from the IL.^50–53^ Overall, this suggests that co-deposition of coextracted elements with REEs may occur due to overlapping reduction potentials. While XPS indicates changes in the surface chemical state, definitive confirmation of alloy or intermetallic formation requires complementary structural characterization.

**Figure 3:**
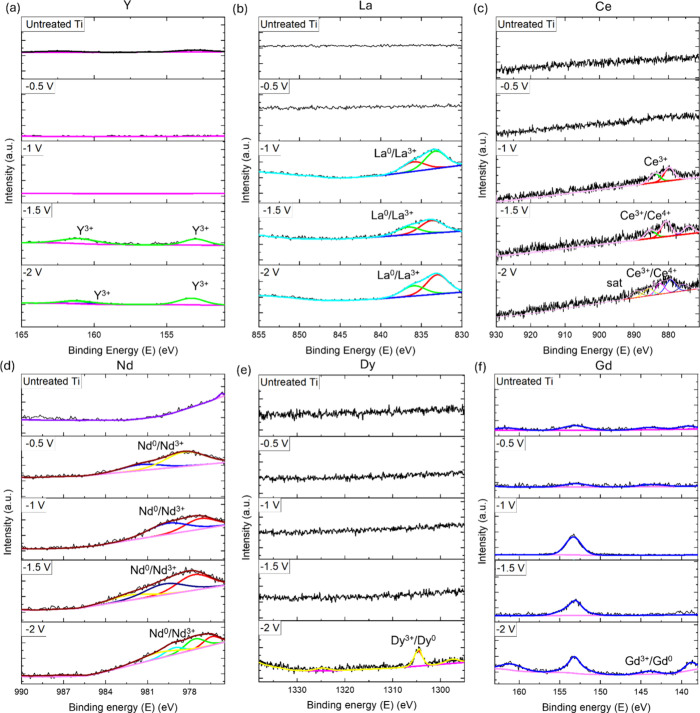
Elemental characterization using X-ray photoelectron spectroscopy after deposition at −0.5, −1.0, −1.5, and −2.0 V vs Pt QRE for 24 h. Deposited REEs were Y (a), La (b), Ce (c), Nd (d), Dy (e), and Gd (f). Untreated Ti foil was taken as a control sample. QRE: quasi-reference electrode.

To investigate the recovery of individual REEs (Y, La, Ce, Nd, Gd, Dy, Ho, and Er), electrochemical deposition was conducted on Ti working electrodes for 0.5, 1, 3, 6, 12, and 24 h. As shown in Figure 4, the recovery profiles of REEs from the REE-enriched IL exhibit distinct dependencies on both the deposition potential and time. Recovery increased progressively with longer deposition durations and more negative potentials, reaching a maximum at −2.0 V after 24 h, corresponding to the potential range where most REE^3+^ ions are reduced to their metallic or oxide forms. Among all REEs, Nd showed the highest recovery, with noticeable deposition even at −0.5 V. In contrast, Dy, Gd, Ho, and Er showed measurable recovery only beyond −1.5 V, reaching 8–12% after 24 h at −2.0 V. Y, La, and Ce exhibited deposition at −2.0 V for shorter durations (starting from 6 h), with additional deposition observed at −1.5 and −1.0 V over longer time intervals. This behavior can be attributed to the low reduction potential of Y, the coexistence of Ce^3+^/Ce^4+^ redox states, and the weak redox activity and low ionic liquid affinity of La.^54,55^ Although recovery increased with longer deposition times, the overall rate gradually decreased, likely because electron transfer slowed while competing side reactions such as oxygen reduction and the formation of hydrogen or hydroxides from residual water became more prominent.^56–58^ Furthermore, a mass balance was also performed to quantify the distribution of REEs deposited on the Ti electrode, retained in the IL, and lost during the recovery process (Figures S8–S11). The results indicate minimal loss, implying that most of the unrecovered REEs remained within the ionic liquid, with less than 10% loss likely arising from reversible deposition, mechanical loss during Ti foil removal, or adsorption of REEs onto the electrochemical cell walls. The co-extracted elements, including Al, Fe, Ni, and Se, were also examined, as shown in Figure S12. These additional co-deposited elements exhibited minimal recovery, contributing to less than 0.25% recovery. However, because these species were present at substantially higher concentrations in the ionic liquid than the REEs, even limited co-deposition can contribute appreciably to the overall current during electrodeposition, thereby reducing the apparent Coulombic efficiency of REE recovery.

**Figure 4:**
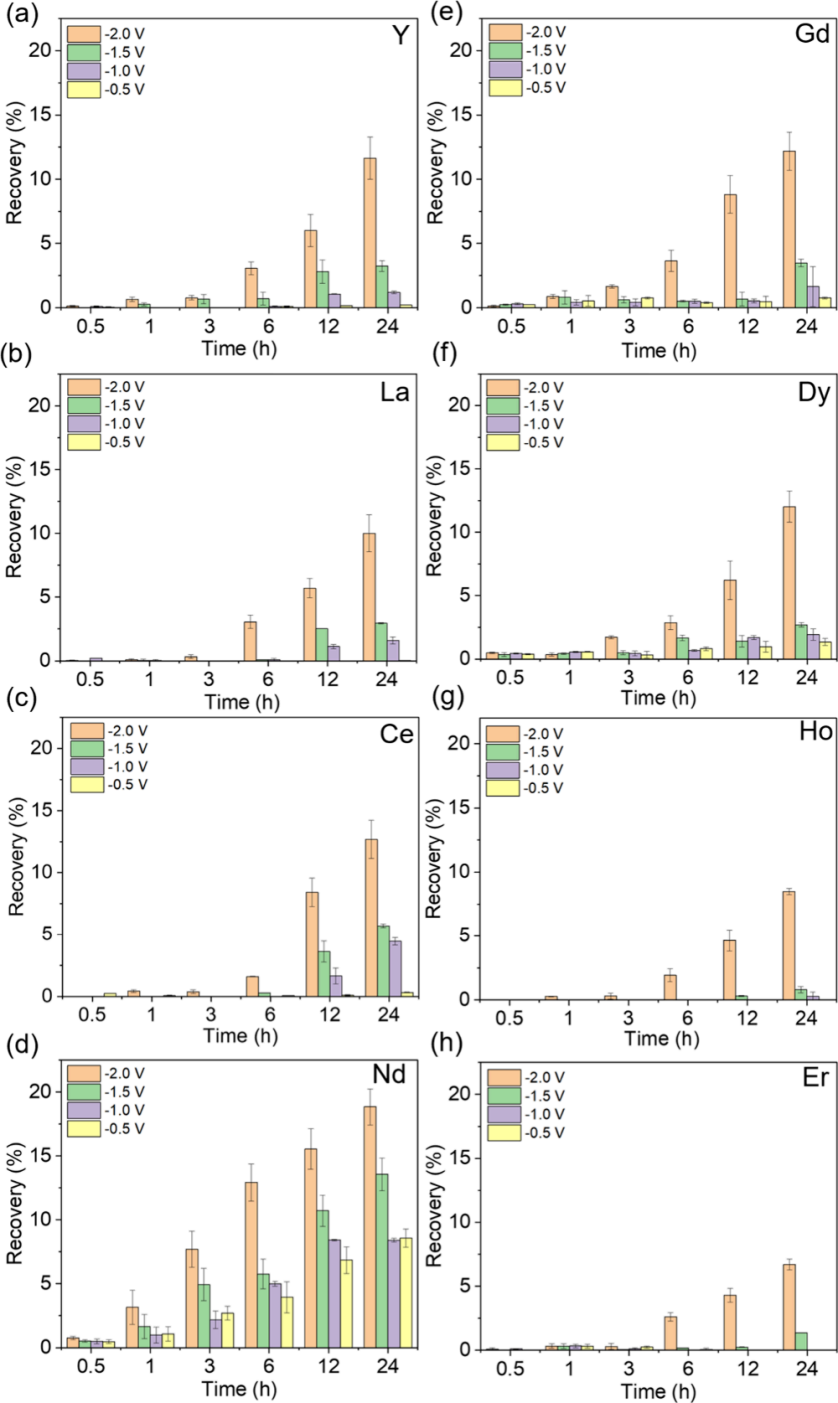
REE recovery from the REEs-enriched IL was evaluated over 0.5–24 h across potentials ranging from −0.5 V vs Pt QRE to −2.0 V vs Pt QRE for Y (a), La (b), Ce (c), Nd (d), Gd (e), Dy (f), Ho (g), and Er (h). QRE: quasi-reference electrode. Data are presented as the mean ± standard deviation (*n* = 3). Error bars indicate the standard deviation from measurement replicates.

To maximize REEs recovery and potential-dependent preferential recovery of Nd, multistep electrochemical deposition was performed from the REE-enriched IL at −0.5 to −2.0 V vs Pt QRE for 6 h (Figures 5a and S13). The 6 h duration was selected as a representative steady-state condition, although a small increase in the recovery continued beyond this time. After each step at −2.0 V, the Ti working electrode was retrieved, rinsed with HNO_3_, and analyzed by ICP-MS. This process was repeated for up to seven steps, with each step progressively depleting REEs from the solution. By the seventh step, the IL became cloudy and formed a gel-like precipitate; therefore, no additional cycle was conducted. The first deposition cycle resulted in a Nd recovery of about 13%, whereas subsequent cycles exhibited a gradual decrease, likely due to slow electron transfer, hydroxyl ion formation, and hydrogen evolution. Furthermore, the [Tf_2_N]^−^ anion may also contribute to the formation and stabilization of the interfacial layer on the electrode surface.^59–62^ Stepwise deposition at −0.5 and −1.0 V did not induce precipitation, indicating stable electrochemical behavior under these conditions. In contrast, higher overall recovery was achieved at −1.5 and −2.0 V, although gel-like precipitation appeared after seven cycles, suggesting IL instability at prolonged, highly negative potentials, consistent with previous reports showing that IL degradation and stability shifts occur primarily under strongly reducing conditions.^59,63^ Furthermore, Nd selectivity was assessed by using the separation factor (β and β_overall_), derived from the recovery ratio of Nd to other REEs at each applied potential. As summarized in Tables S3 and S4, higher β_overall_ values indicate more effective separation, with the maximum β_overall_ for Nd observed at −0.5 V. At this potential, Nd exhibited ∼25% recovery with a β_overall_ of 37, while other REEs showed negligible deposition. Reducing the potential to −1.0 V resulted in additional recovery of Gd and Dy alongside Nd, with a β_overall_ of 11. Further reduction to −1.5 and −2.0 V facilitated the deposition of Er and other heavier REEs. At −1.5 V, Nd recovery reached 31.9% (β_overall_, 11), and at −2.0 V, total Nd recovery exceeded 48%, with other REEs recovering between 10 and 18% (β_overall_, 5). As shown in Figure 5a,b, reducing the potential enhanced overall REE recovery but reduced the separation factor, reflecting a trade-off between selectivity and total deposition yield.^62,66^ At increasingly negative potentials, enhanced reduction kinetics and competing side reactions likely diminish selectivity by promoting co-deposition of other metals and nonspecific surface coverage, resulting in diminished selectivity and a decrease in the β_overall_.

**Figure 5:**
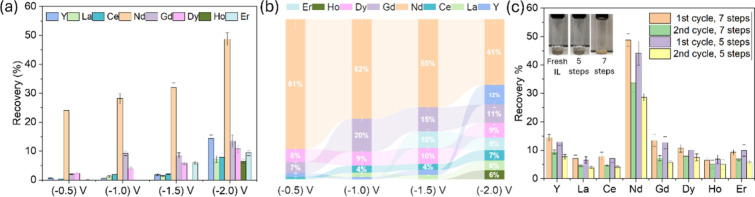
Recovery percentages of REEs after seven sequential deposition steps at potentials ranging from −0.5 to −2 V vs Pt QRE for 6 h (a). Relative abundance (%) of REEs at different potentials after seven deposition steps (b). Recyclability of the IL for electrochemical recovery, comparing the performance of fresh IL with regenerated IL after the fifth and seventh steps (c). QRE: quasi-reference electrode.

To examine possible mechanistic changes in the IL following the seventh electrodeposition step in the gel-like precipitation form, proton (^1H) NMR spectroscopy was carried out to evaluate any structural alterations. As shown in Figure S14, the red spectrum represents the REE-enriched IL prior to electrochemical deposition, while the blue spectrum corresponds to the IL after the seventh deposition step. Both spectra exhibited two distinct sets of proton peaks (labeled 1, 2, and A, B). In the electrochemically treated IL, a slight downfield shift was observed, indicating that electron transfer likely occurred during deposition.^64^ The absence of new peaks in the treated IL suggests that the IL in solution form did not undergo significant chemical degradation or decomposition. To examine the [TF_2_N]^−^ stability, XPS on the gel-like IL was also performed after the seventh electrochemical cycle (Figure S15). Both the REEs-enriched IL and the electrochemically treated IL showed a characteristic F peak at 688.6 eV, corresponding to the CF_3_ group of the [Tf_2_N]^−^ anion. After the seventh electrochemical cycle, the appearance of an additional peak at 685.5 eV suggests partial degradation of the [Tf_2_N]^−^ anion during prolonged electrochemical operation, likely promoted by trace water, resulting in the formation of fluoride-containing species. This is consistent with previous reports showing that the [Tf_2_N]^−^ anion can generate F^–^ ions under highly negative potentials.^59,63^ These considerations define the current boundaries of recyclability and inform future optimization of the IL lifetime and regeneration strategies.

To reuse the IL after stepwise electrodeposition at −2.0 V, regeneration was performed after the seventh deposition cycle, where the IL exhibited a cloudy appearance. As a control, regeneration was also performed after the fifth step, during which no visible changes were observed in the IL. The regeneration process involved washing with an aqueous phase, followed by stripping with 1.5 M HCl to remove residual REEs and impurities. The regenerated IL was subsequently reused for REE leaching from CFA, demonstrating extraction performance comparable to that of the fresh IL (Figure S16). Next, it was again used for stepwise electrochemical deposition (Figure 5c). A slight decrease in recovery efficiency was observed upon reuse, likely due to the mass loss of the REE-enriched IL during regeneration. Although regeneration requires dilute acid treatment, which may result in additional chemical inputs, the process reduces solvent consumption relative to single-use operation. Nevertheless, even after recycling, the IL retained its ability to be regenerated and reused for electrochemical deposition with minimal change in performance, indicating its sustainable and reusable nature for REE recovery.

### Projected Environmental Impact and Circularity Potential

From an environmental perspective, the [Hbet]­[Tf_2_N] IL-based electrochemical process incorporates features such as reduced hazardous reagent use, limited water demand, and solvent recyclability, which collectively lower process-related environmental burdens. Unlike conventional acid leaching, such as those employing hydrochloric, nitric, or sulfuric acids, which generate acidic effluents containing heavy metals that require neutralization and disposal.^65,66^ The IL-based approach in this study produced negligible liquid waste and avoided the use of strong acids. It also enabled potential-dependent preferential electrodeposition of Nd over major CFA constituents, thereby reducing secondary waste generation. Furthermore, the demonstrated regeneration and reuse of the IL across multiple electrochemical cycles establishes a closed-loop process that substantially reduces the environmental footprint relative to conventional single-use extraction systems. However, comprehensive assessment and quantitative evaluation of the regeneration efficiency and long-term sustainability of the IL system are still needed in the future. Previous life cycle assessment (LCA) studies have validated the environmental benefits of the electrochemical process, highlighting its reduced chemical consumption, solvent recyclability, and elimination of water use, which together enable near-zero wastewater discharge and minimal solid waste generation.^67^ The current IL-based approach operated with very low voltage compared to conventional acid-leaching and molten-salt electrorefining methods, which typically require higher temperatures and sustained high current densities. Using just 1 g (≈1 mL) of [Hbet]­[Tf_2_N] per campaign and regenerating it after each 7-step cycle reduced solvent costs by nearly 86% compared to a single-use operation. The most economically favorable condition was found at −0.5 V, achieving a separation factor of 37 under laboratory-scale operation. Additionally, based on a preliminary estimate considering only the electrical energy input for electrodeposition, the global warming potential (GWP) was calculated to be ∼22.7 kg CO__2__e per kg Nd using an emission factor of 0.394 kg CO__2__e kWh^–1^.^68^ This estimate is lower than reported emissions for REE extraction from clays using leaching (258–408 kg CO__2__e per kg rare earth oxides).^69^ This comparison is not directly equivalent due to differences in system boundaries. The GWP reported in this study represents a lower-bound estimate, as it accounts only for the electrodeposition step and excludes upstream and downstream contributions, such as ionic liquid synthesis, heating, separation, and regeneration. In contrast, literature values for REE production typically encompass the full process chain, including mining, leaching, and purification. Therefore, this comparison is intended to provide an order-of-magnitude context rather than a direct quantitative benchmark. While the current results remain modest at the laboratory scale, the modular design of the system and the potential for solvent recyclability suggest a pathway toward scalability, although substantial improvements in charge efficiency, mass transport, and overall energy performance will be required for practical implementation. Collectively, these characteristics point toward an environmentally friendly and circular recovery pathway, although further improvements in efficiency and process optimization are required prior to practical implementation. While the IL system enables reduced solvent usage and offers recyclability, its overall environmental impact requires careful evaluation. IL degradation during repeated electrochemical operations may introduce additional considerations that must be taken into account in assessing the process footprint.

## Discussion

This work introduces an integrated approach for REE recovery from CFA using a [Hbet]­[Tf_2_N] IL that functions dually as both extractant and electrolyte. This dual functionality enables efficient REE leaching and electrochemical deposition under nonaqueous conditions within the IL’s wide electrochemical stability window. Electrochemical deposition experiments were conducted between −0.5 and −2.0 V (vs Pt QRE), revealing potential-dependent preferential electrodeposition of Nd at −0.5 V, while comprehensive REE recovery was achieved at −2.0 V. Multistep deposition at −2.0 V yielded the highest cumulative recovery, demonstrating tunability between potential-dependent preferential deposition and total yield. These findings highlight that potential control can serve as a precise tuning parameter for potential-dependent preferential REE recovery, validating the [Hbet]­[Tf_2_N]-based electrochemical system as a viable route for element-specific separation from complex matrices such as CFA. Following electrochemical operation, the IL was successfully regenerated and reused for subsequent recovery cycles, confirming its chemical stability and recyclability. After seven electrodeposition cycles at lower negative potentials, slight precipitation was observed in the ionic liquid; however, its functional properties were retained, and the ionic liquid could be regenerated and reused for further REE recovery. The recovered REEs remained primarily in solution form, indicating that subsequent separation through complementary techniques, such as chromatographic separation, ion-selective extraction, or electrodeposition under optimized mass-transfer conditions, can be explored in future studies. Nd in alloy mixture can be separated through a combination of magnetic separation and oxalate precipitation, providing a feasible pathway for pilot-scale implementation. The current limitation arises from slow electron-transfer kinetics at the IL–electrode interface, which restricts complete metal reduction at extended times. From both environmental and economic perspectives, our IL-based approach provides an environmentally friendly alternative to conventional acid leaching methods by eliminating hazardous reagents, minimizing waste generation, and enabling solvent regeneration. Conventional REE recovery typically relies on large volumes of concentrated acids during primary leaching and purification. In contrast, acid is not used in the primary extraction step of the present process but is applied only at low concentration during ionic liquid regeneration. While the system demonstrates the dual functionality of the IL as both extractant and electrolyte, along with potential-dependent preferential recovery and IL reusability, improvements in energy efficiency and operating conditions are needed before scalable implementation. In particular, the current low Coulombic efficiency (<2%) and extended deposition times result in higher energy consumption per unit recovery (57.6 kWh/kg), reflecting mass transport limitations in the viscous ionic liquid. In this context, the present study is positioned as an early-stage proof-of-concept that demonstrates feasibility rather than a process-ready system. Existing electrochemical approaches, particularly high-temperature molten salt systems, have reported Coulombic efficiencies of ∼80–85% with specific energy consumption (SEC) of ∼2–8 kWh kg^–1^, but these require operation at elevated temperatures (∼500–800 °C) under inert atmospheres.^70,71^ Furthermore, hydrometallurgical processes achieve high recovery yields but involve multistep flowsheets with varying temperature conditions and significant solvent and chemical requirements, making their overall energy and process complexity highly system dependent.^72^ Additionally, emerging methods such as flash Joule heating report low energy consumption (∼1 kWh kg^–1^) but operate at extreme transient temperatures (>3000 °C) and are primarily limited to pretreatment rather than direct separation.^73^ Taken together, these differences highlight that direct quantitative comparison across these approaches is not straightforward, as each involves distinct process steps, system boundaries, and energy inputs. Therefore, the comparison presented here is intended to provide an order-of-magnitude context rather than a direct performance benchmark. Future scale-up will require systematic evaluation of electrode fouling, ionic liquid viscosity-driven mass-transfer limitations, and continuous-flow electrochemical systems, as well as benchmarking against industrial electrowinning metrics. Future research can aim to enhance REE recovery with greater energy efficiency and extend this approach to other secondary waste sources, such as electronic waste, industrial wastewater, and medical residues. Additionally, the [Hbet]­[Tf_2_N] IL can be further optimized to achieve higher recovery yields, high Coulombic efficiency, and improved energy efficiency while maintaining chemical stability and minimizing degradation.

Future work may also focus on enhancing recovery performance by suppressing excessive electric double-layer formation, improving interfacial mass transport, and reducing ionic liquid viscosity to facilitate faster ion mobility and transport. Additionally, while this work establishes a proof-of-concept for tunable electrochemical separation, additional optimization and system-level integration will be required to achieve robust process-scale selectivity. These findings underscore that continued process optimization—particularly in improving charge efficiency, mass transport, and energy performance—remains a critical priority for future development.

Overall, this study offers a new paradigm for REEs recovery from real CFA samples, demonstrating the first use of a single [Hbet]­[Tf_2_N] IL as both an extractant and electrolyte. The developed closed-loop, IL-mediated electrochemical platform promotes sustainable materials circularity and strengthens critical element supply resilience, providing essential guidance for the design of next-generation recovery systems from secondary waste resources.

## Methods

### Materials

[Hbet]­[Tf_2_N] salts (99%) were obtained from IOLITEC Ionic Liquids technologies. Fly ash 93927 was derived from a power plant burning Class C PRB coal. Ti foil as the working electrode and Pt as the reference and counter electrode were bought from McMaster and StonyLab. IPA and acetone were purchased from VWR.^74,75^

### Characterization

NMR samples were prepared by dissolution of the sample in deuterated DMSO and filled in an NMR tube. Measurements were performed on a Bruker Avance Neo WB 300 MHz NMR spectrometer at resonance frequencies of 300 MHz for ^1^H and 75.5 MHz for proton spectra. The surface morphology of the electrodeposits was evaluated by using a SEM Hitachi SU-8230 and atomic force microscopy (AFM, Bruker), and using AppNano ACT tapping mode AFM probes from Applied Nanosciences. Chemical composition of the sample surfaces was analyzed by X-ray photoelectron spectroscopy characterization on REEs deposited Ti foil (XPS, Thermo Fisher Scientific K-Alpha XPS) with a 400 μm microfocused monochromatic Al Kα X-ray source, which has an analysis depth of less than 5 nm. F XPS characterization after the seventh cycle of electrochemical treatment was done in powder form.

### Leaching

Briefly, CFA, water-saturated IL, and an aqueous (AQ) solution containing 1.0 M NaNO_3_ were combined in a small vial to maintain an IL/AQ mass ratio of 1:1 and a solid-to-total-liquid ratio of 15:1 (mg/g). NaNO_3_ was added to promote phase separation between the IL and AQ layers.^26^ The mixture was heated with magnetic stirring at 85 °C in an oil bath for 3 h. After being cooled to room temperature, the suspension was allowed to stand undisturbed at room temperature until complete phase separation was observed (two visually distinct layers). The upper aqueous phase was removed by careful pipetting (or decanting) while minimizing disturbance of the interfacial region, and the IL phase was retained for subsequent steps.^25,28^ The filtered AQ and IL phases were collected in a new vial, where 10 mg betaine/g AQ and 25 mM ascorbic acid were added to the AQ phase to further enhance the REE distribution in the IL phase and minimize iron coextraction.^25,26^ The mixture was again heated with magnetic stirring at 85 °C in an oil bath for 1.5 h. Following cooling, the AQ phase was separated and analyzed by inductively coupled plasma-optical emission spectrometry (ICP-OES), and the IL phase was collected for subsequent stripping or electrochemical deposition.

### Stripping

Briefly, an aqueous 1.5 M HCl solution was added to the IL-containing vial at an IL/HCl mass ratio of 1:1. The mixture was heated with magnetic stirring at 85 °C for 1.5 h, then cooled to room temperature, and stored at 4 °C overnight. After the mixture was cooled, the HCl aqueous phase was separated and analyzed by ICP-OES.

### Electrochemical Techniques

LSV measurements were performed on a Biologic potentiostat in a three-electrode electrochemical cell with a Ti foil working electrode (width 0.5 ± 0.2 cm), a platinum plate as the counter electrode, and a platinum wire as the reference electrode in the range of 0 to −2.5 V and with a scan rate of 20 mV s^–1^ at 80 °C. Prior to the measurement, the platinum electrodes were rinsed with acetone or ethanol and cleaned with fuzz-free tissue before drying in air. The working electrode was cleaned with sonication in soapy water, IPA, and acetone for 10 min each before a final rinse with water and dried in air. For Fc/Fc^+^ calibration, a cyclic voltammogram was recorded using a three-electrode setup in a 7 mM ferrocene solution, with a glassy carbon working electrode and platinum serving as both the reference and counter electrodes, at a scan rate of 20 mV s^–1^. REEs electrodeposition was conducted at −2.0, −1.5, −1.0, and −0.5 V under ambient conditions for durations ranging from 15 min to 24 h in a 1 mL solution. To enhance mass transfer and reduce the viscosity of the IL, experiments were performed at 80 °C and 300 rpm. The electrodeposits on the Ti working electrode were dissolved in 3% nitric acid and quantified using ICP-OES/ICP-MS. Remaining REEs in the IL were extracted by vortexing for 1 min, followed by 1 h of sonication in 3% nitric acid prior to analysis. Recovery was calculated using the deposited mass divided by the initial mass. Nd selectivity was evaluated using the separation factor (β), calculated from the relative recovery of Nd compared to other individual REE at each applied potential, as shown in eq 1. In addition, an overall separation factor (β_overall_) was determined to represent the selectivity of Nd with respect to the total REEs recovery at each potential, as shown in eq 2. The separation factor was defined as

$$\beta =\frac{D_{\mathrm{Nd}}}{D_{\mathrm{REE}}}$$ 1

$$\beta_{\mathrm{overall}}=\frac{D_{\mathrm{Nd}}}{D_{\mathrm{Total}\;\mathrm{REEs}}}$$ 2

where *D*_Nd_ represents the recovery percentage of Nd, *D*_REE_ denotes the recovery percentage of a given rare earth element, and *D*_Total REEs_ corresponds to the overall recovery of all other REEs (excluding Nd), determined as the ratio of the total mass of other REEs deposited on the electrode to their total initial mass in the feed solution.

Columbic efficiency (η_c_) was calculated using

$$\eta_{c}=\frac{Q_{\mathrm{theoretical}}}{Q_{\mathrm{applied}}}\times 100$$ 3

where *Q*_theoretical_ represents the charge required for metal deposition based on Faraday’s law and *Q*_applied_ is the total charge passed during electrochemical deposition, calculated from the applied current (A) and deposition time (s). SEC was calculated using

$$\mathrm{SEC}\left(\frac{\mathrm{kWh}}{\mathrm{kg}}\right)=\frac{E_{\mathrm{Consumed}}(\mathrm{kWh})}{m_{\mathrm{Nd}}(\mathrm{kg})}$$ 4

where *E*_consumed_ is the total energy consumed during the electrodeposition, and *m*_Nd_ is the mass of Nd recovered, quantified using ICP-MS.

GWP was calculated using

$$\mathrm{GWP}=E_{\mathrm{Consumed}}(\mathrm{kWh})\times \mathrm{Emission}\;\mathrm{factor}\left(\mathrm{kg}\frac{{\mathrm{CO}}_{2}e}{\mathrm{kWh}}\right)$$ 5

where *E*_consumed_ is the total energy consumed during the electrodeposition, and emission factor is 0.394 kg CO_2_e/kWh, determined from an emission factor value from the United States Environmental Protection Agency.^68^

Statistical significance was determined using one-way ANOVA followed by Tukey’s post hoc test (*p < 0.05, **p < 0.01, ***p < 0.001, ***p < 0.0001).

## Supplementary Material



## Notes

Scheme of REEs extraction from CFA, REE concentration in 93927 CFAsample, composition of major and trace REE elements, cyclic voltammogram of thethree-electrode electrochemical system in ferrocene solution, standardelectrochemical potential of different elements, optical images of Ti foils, average surface roughness, XPS spectra, mass balance, recovery (%) andseparation factor, ^1^H NMR spectra, and regeneration of IL after 5steps and 7 steps electrochemical recovery (PDF)
