# Supplementary material for: Electrochemical Recovery of Rare-Earth Elements from Coal Fly Ash Using Ionic Liquids as both Extractant and Electrolyte
Source: Environ Sci Technol. 2026 May 27;60(22):16245–56. doi: 10.1021/acs.est.5c16688 (PMC13261875; doi:10.1021/acs.est.5c16688)
Supplement: Supplementary file 1 [file es5c16688_si_001.pdf]

Supporting Information

Electrochemical Recovery of Rare Earth Elements from Coal Fly Ash Using Ionic Liquids as both Extractant and Electrolyte

Anuja Tripathi[1], Ting Liu[1], Joe F. Bozeman III[1,2], Ching-Hua Huang[1]*, Xing Xie[1]*

[1]School of Civil and Environmental Engineering, Georgia Institute of Technology, 311 Ferst Drive NW, Atlanta, GA, 30332, USA

[2]Jimmy and Rosalynn School of Public Policy, Georgia Institute of Technology, 258 4th St NW, Atlanta, GA 30332, USA

*Corresponding authors-email: xing.xie@ce.gatech.edu ching-hua.huang@ce.gatech.edu

This Supporting Information contains 18 pages, 4 tables, and 16 figures.

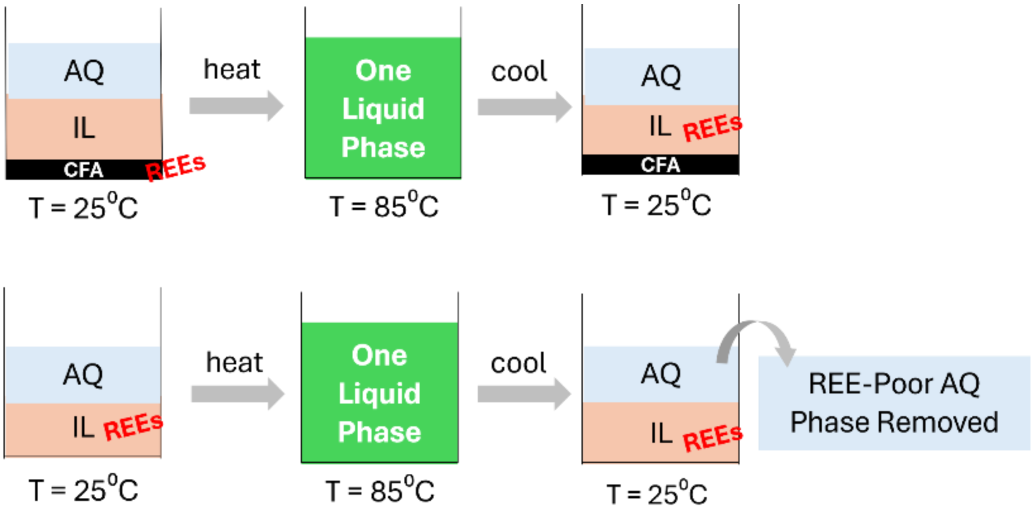

Figure S1: Scheme of REEs extraction from CFA using [Hbet][Tf_2N] ionic liquid.

Table S1: REEs concentration in 93927 CFA sample[1]

REE	ug/g ash
**Sc**	22.13
**Y**	31.52
**La**	46.72
**Ce**	91.09
**Pr**	9.4
**Nd**	33.65
**Sm**	6.52
**Eu**	2.52
**Gd**	6.75
**Tb**	0.97
**Dy**	5.52
**Ho**	1.11
**Er**	3.15
**Tm**	0.44
**Yb**	2.81
**Lu**	0.43
**Al**	85827
**Fe**	38142
**Ni**	40
**Se**	5

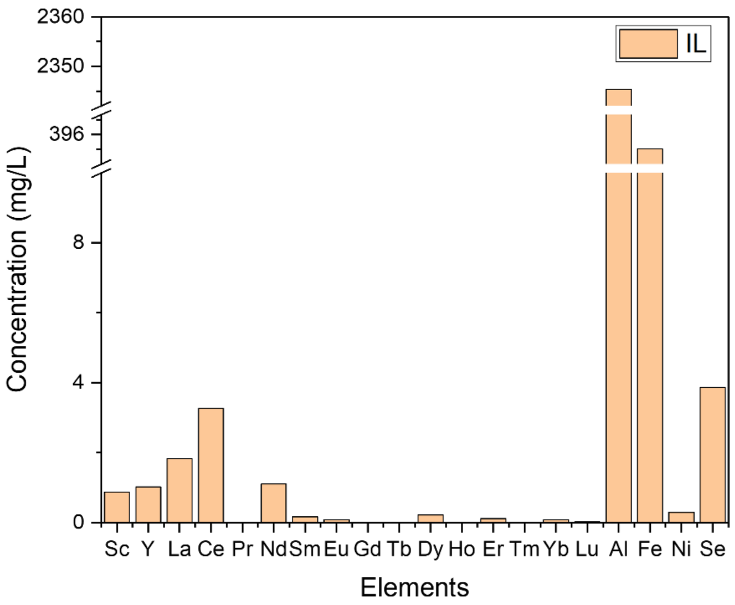

Figure S2: Composition of major and trace REE elements in REEs-enriched ionic liquid extracted from 93927 CFA in this study.

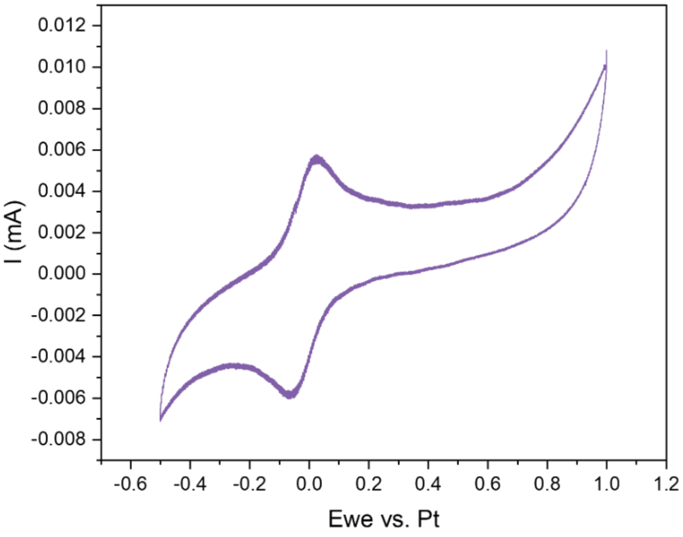

Figure S3: Cyclic voltammogram of the three-electrode electrochemical system recorded in a 7 mM ferrocene solution, using a glassy carbon working electrode with platinum serving as the reference and counter electrodes at a scan rate of 20 mV s^{-1}.

Table S2: Standard electrochemical potential of different elements vs Fc/Fc^+ (Fc/Fc^+ = 0.40 vs. SHE)

Elements	Reduction potential (V vs. Fc/Fc^+)[2–8]
**Se**	-0.9
**Al**	-2.4
**Gd**	-2.4
**Ho**	-2.4
**Y**	-1.95
**Er**	-2.4
**S**	-0.48
**F**	+2.3
**Fe**	-0.5
**La**	-2.4
**Ni**	-0.3/-0.4
**Ce**	-2.3/-2.6
**Nd**	-2.4
**Dy**	-3.5

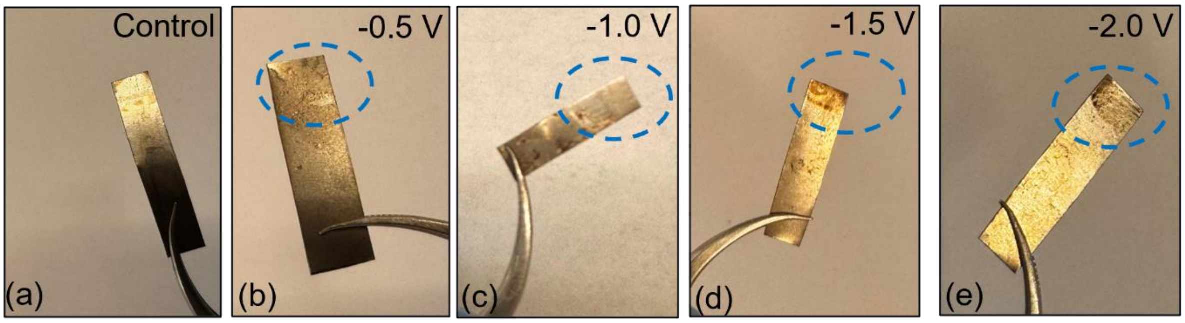

Figure S4: Optical images of Ti foil (a) as control, chronoamperometric depositions for 24 h at -0.5 V vs Pt QRE (b), -1.0 V vs Pt QRE (c), -1.5 V vs Pt QRE (d), and -2.0 V vs Pt QRE (e). QRE: Quasi-reference electrode.

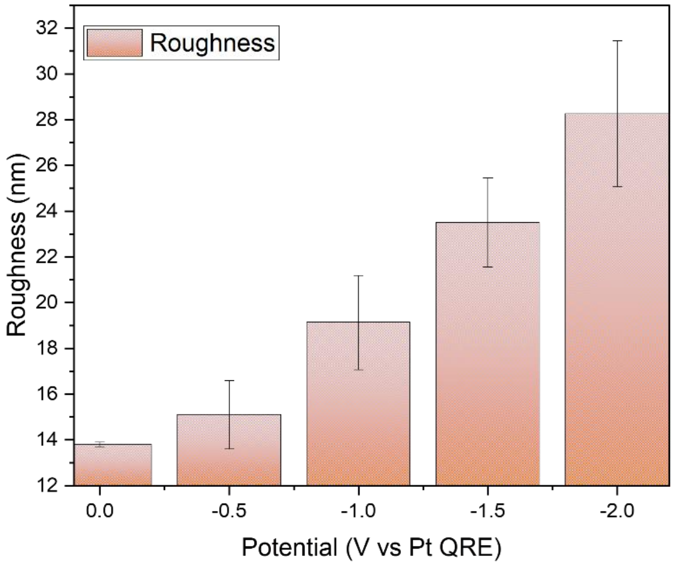

Figure S5: Average surface roughness measured by atomic force microscopy for the control Ti surface after electrodeposition in the potential range of -0.5 V vs Pt QRE to -2.0 V vs Pt QRE for 24 h. QRE: Quasi-reference electrode.

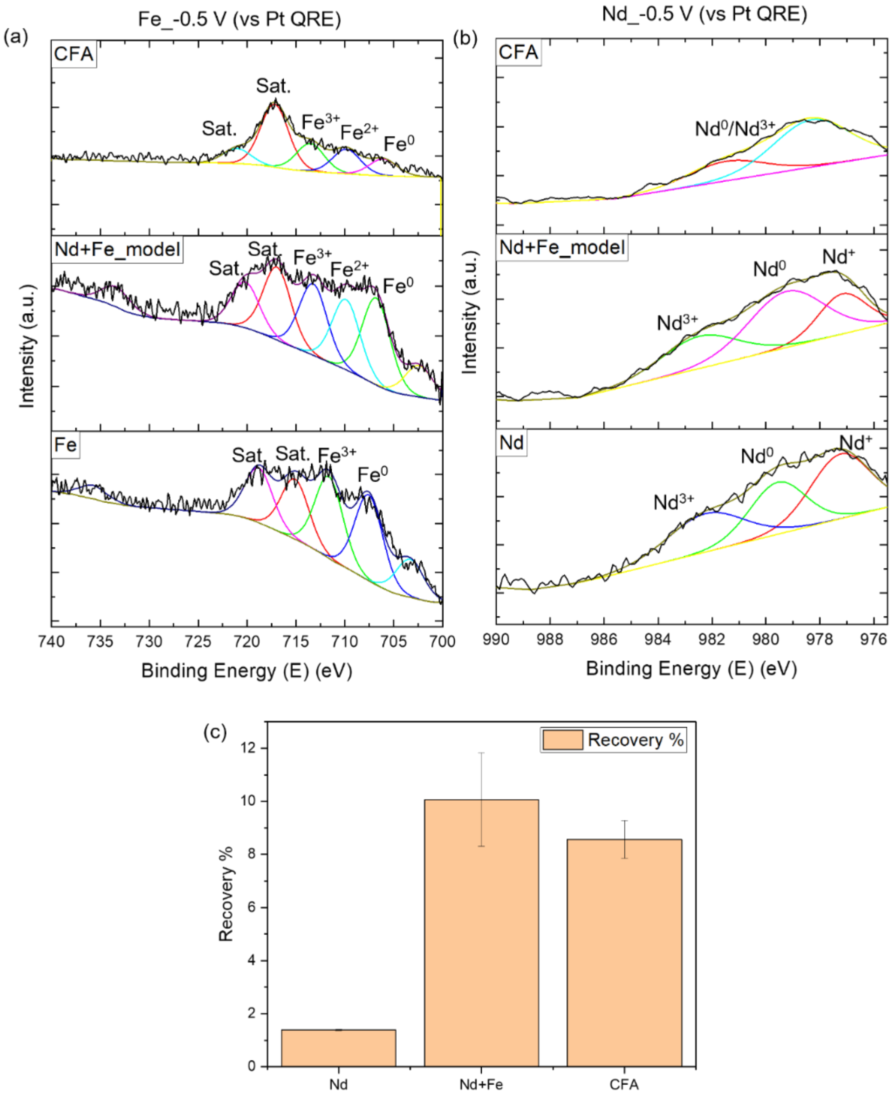

Figure S6: XPS spectra of deposited Fe and Nd (a, b), along with the recovery % of Nd (c) on Ti foil obtained via chronoamperometry at −0.5 V vs Pt quasi-reference electrode (QRE). The deposition behavior of Fe and Nd is compared between a synthetic Nd–Fe mixed electrolyte and coal fly ash (CFA)-derived electrolyte samples.

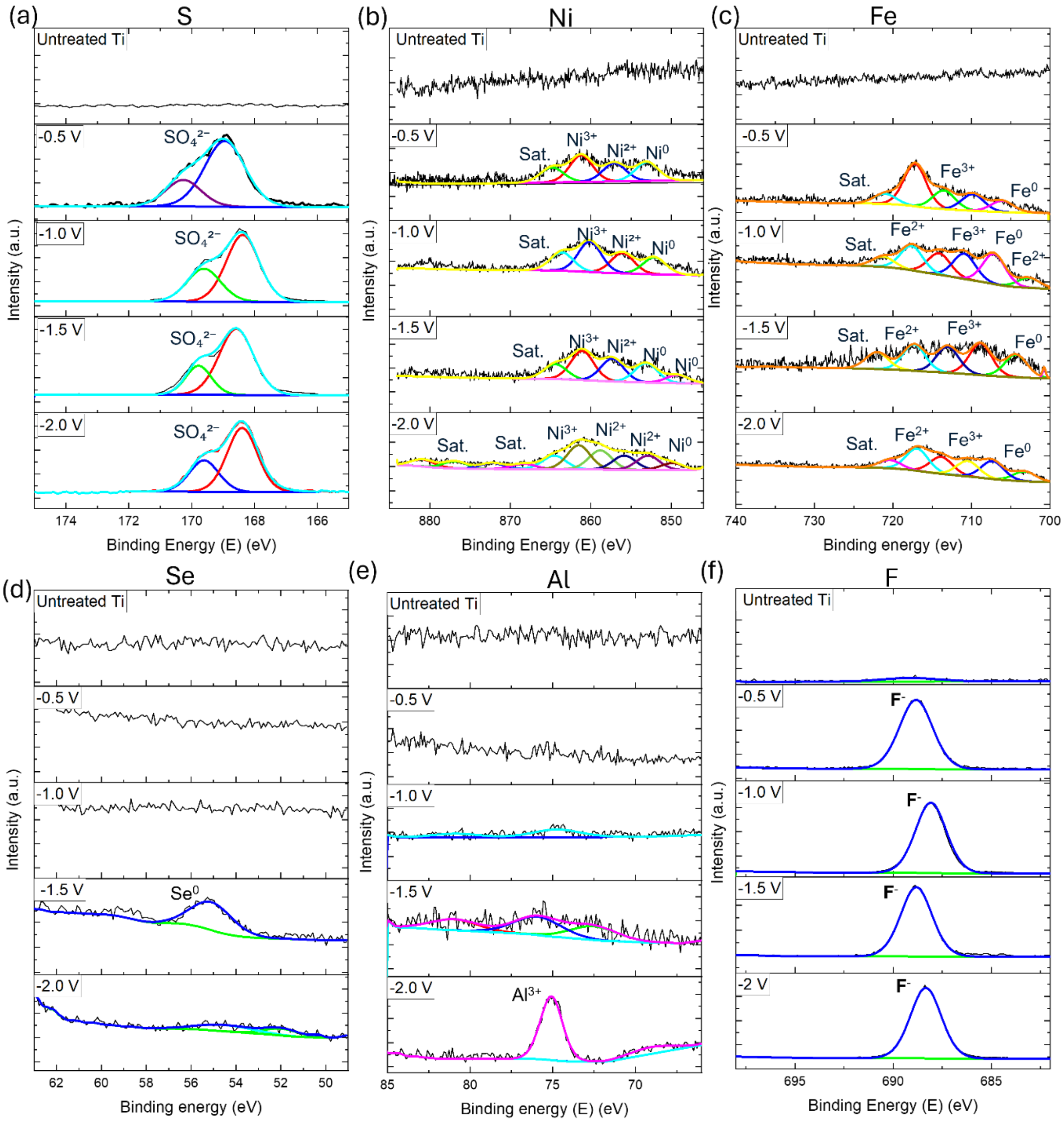

Figure S7: XPS spectra of additional elements deposited on Ti foil using chronoamperometry technique in the potential range of -0.5 V vs Pt QRE to -2.0 V vs Pt QRE for 24 h. Metals recovered are S (a), Ni, (b), Fe(c), Se (d), Al (e), and F (f). QRE: Quasi-reference electrode.

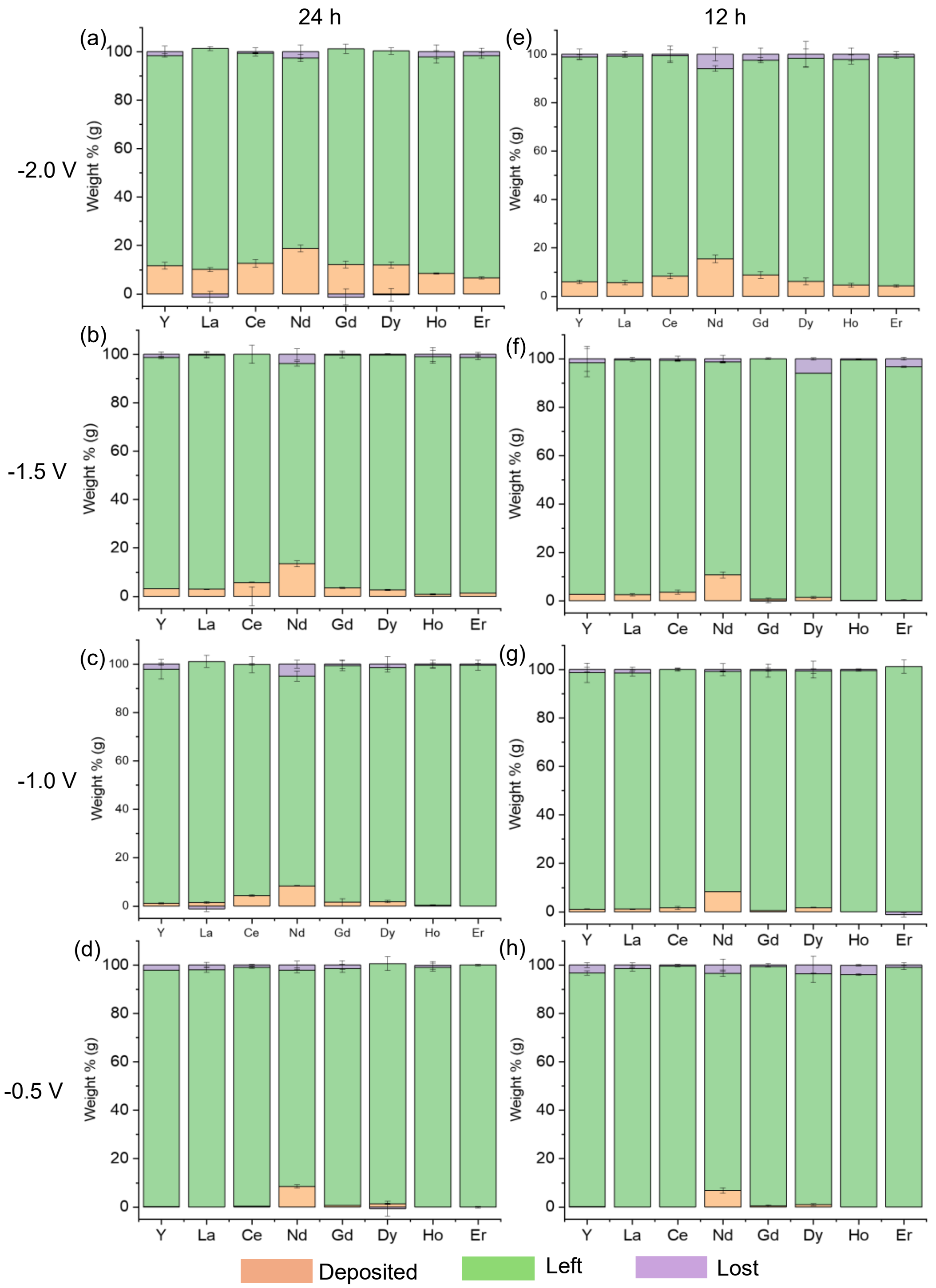

Figure S8: Mass balance of recovered REEs from CFA-extracted in [Hbet][Tf_2N] IL at -2.0 V vs Pt QRE, -1.5 V vs Pt QRE, -1.0 V vs Pt QRE, and -0.5 V vs Pt QRE for various deposition times (12 h and 24 h) using chronoamperometry. Stacked bars show the percentage of REEs, other metals, and impurities in the deposits. Error bars represent standard deviations from replicate measurements. QRE: Quasi-reference electrode.

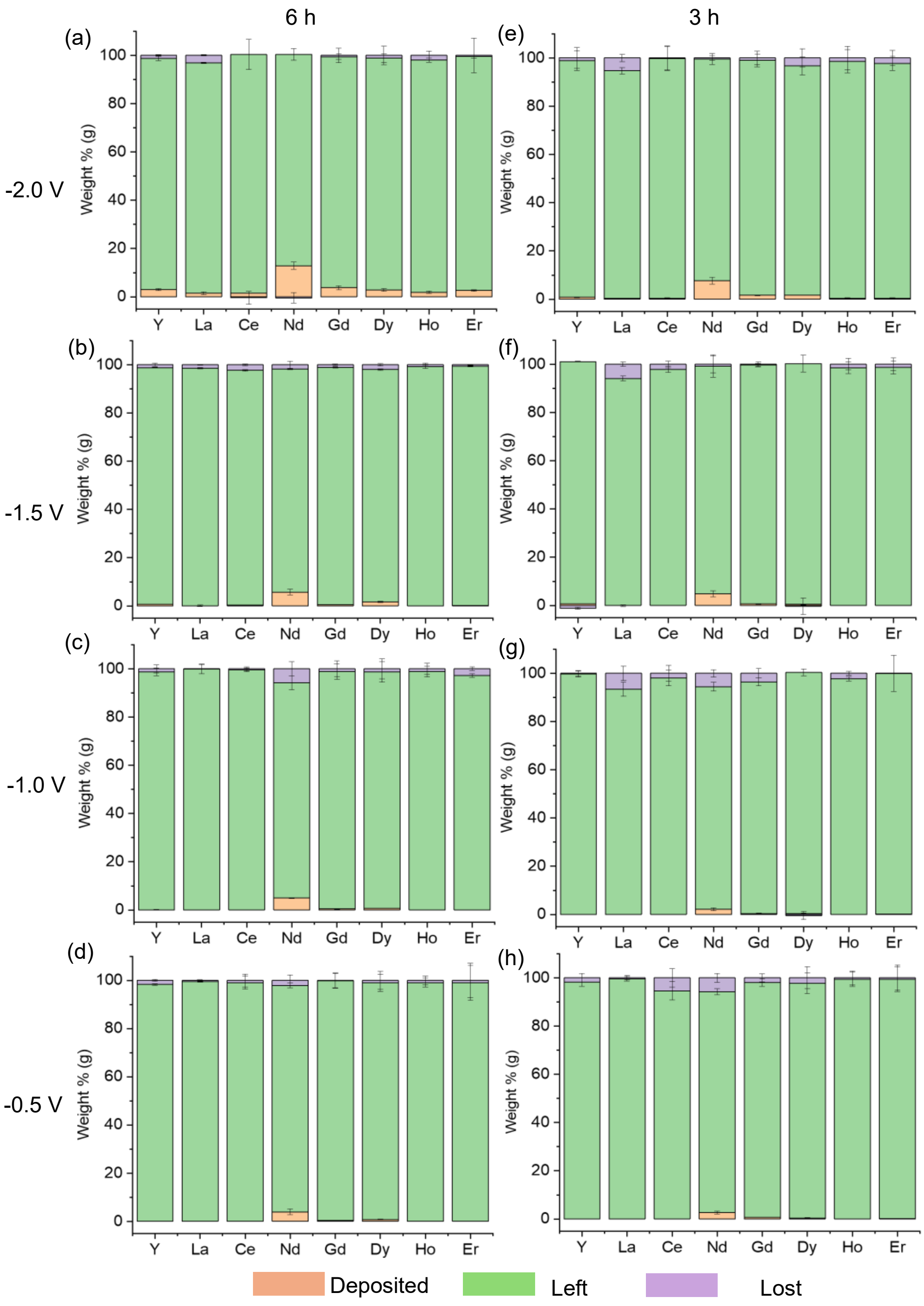

Figure S9: Mass balance of recovered REEs from CFA-extracted in [Hbet][Tf_2N] IL at -2.0 V vs Pt QRE, -1.5 V vs Pt QRE, -1.0 V vs Pt QRE, and -0.5 V vs Pt QRE for various deposition times (3 h and 6 h) using chronoamperometry. Stacked bars show the percentage of REEs, other metals, and impurities in the deposits. Error bars represent standard deviations from replicate measurements. QRE: Quasi-reference electrode.

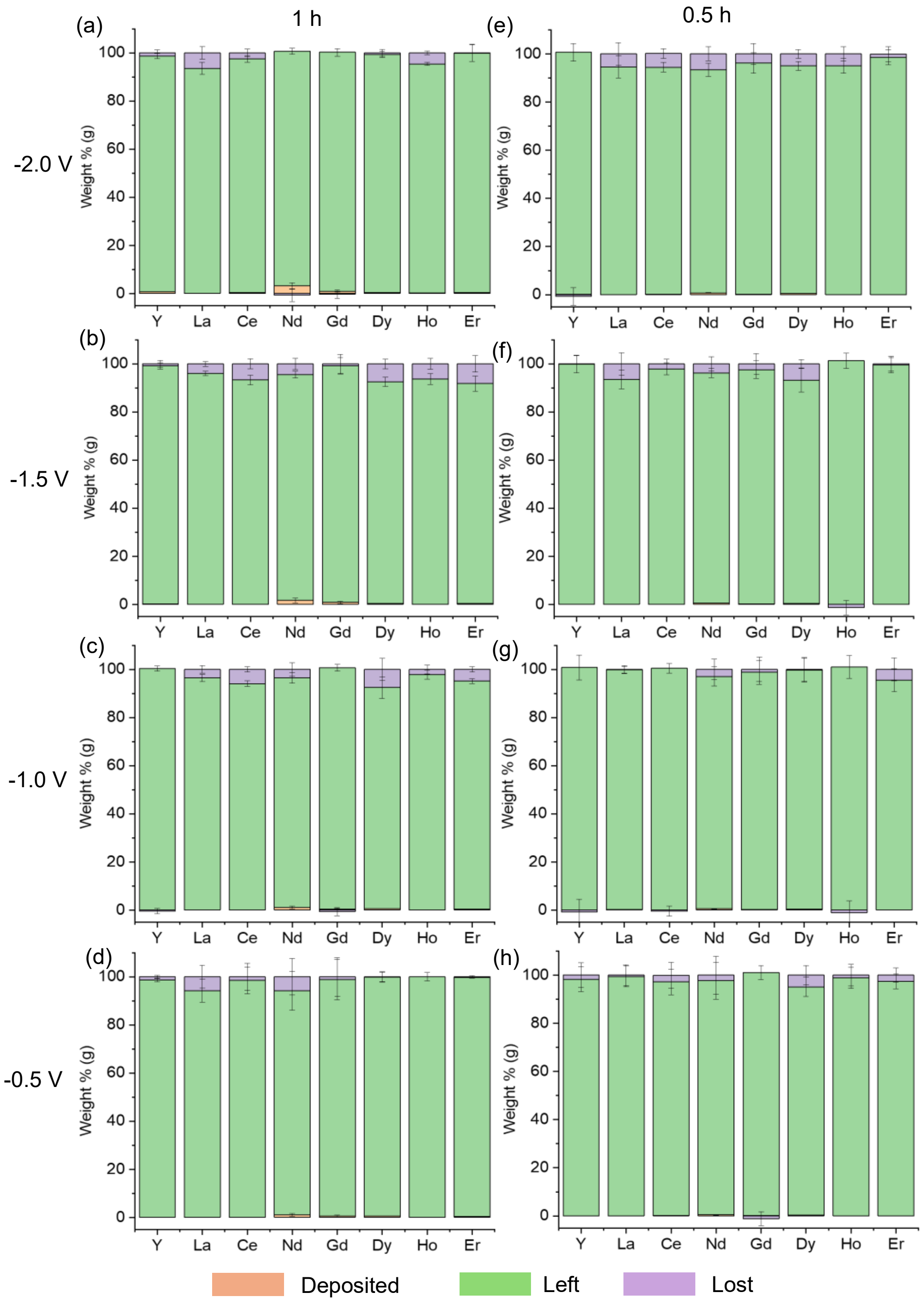

Figure S10: Mass balance of recovered REEs from CFA-extracted in [Hbet][Tf_2N] IL at -2.0 V vs Pt QRE, -1.5 V vs Pt QRE, -1.0 V vs Pt QRE, and -0.5 V vs Pt QRE for various deposition times (1 h and 0.5 h) using chronoamperometry. Stacked bars show the percentage of REEs, other metals, and impurities in the deposits. Error bars represent standard deviations from replicate measurements. QRE: Quasi-reference electrode.

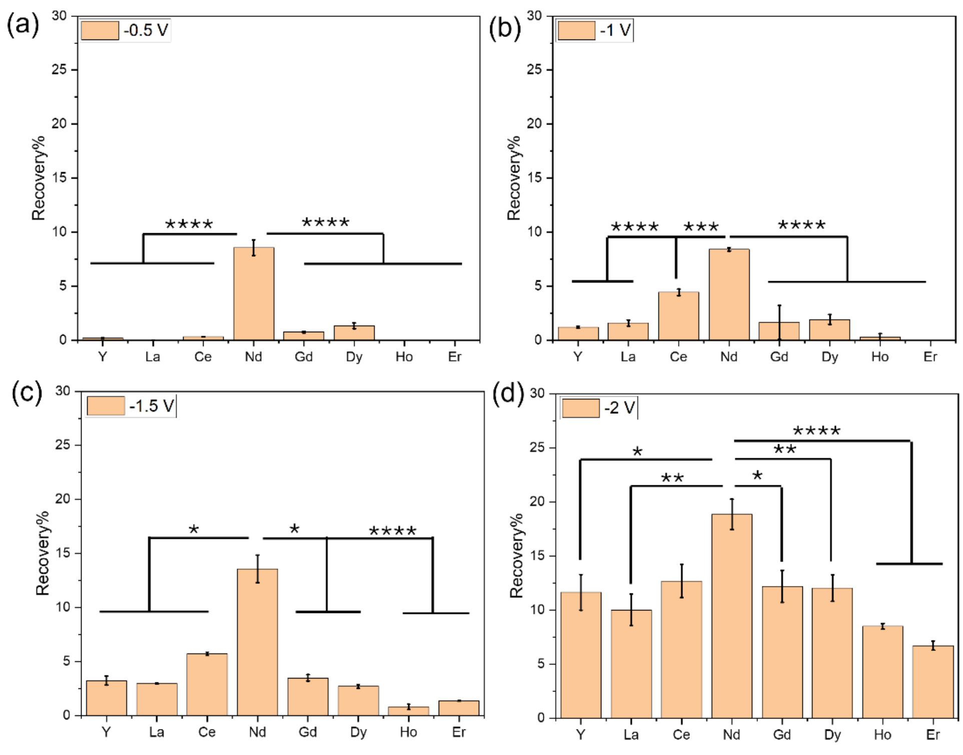

Figure S11: Potential-dependent recovery of REEs during electrochemical deposition. Recovery of Y, La, Ce, Nd, Gd, Dy, Ho, and Er at applied potentials of −0.5 V (a), −1.0 V (b), −1.5 V (c), and −2.0 V (d) (vs Pt quasi-reference electrode). Data represents mean ± SD, n = 3, *p < 0.05, **p < 0.01, ***p < 0.001, ***p < 0.0001. Error bars indicate standard deviation from measurement replicates.

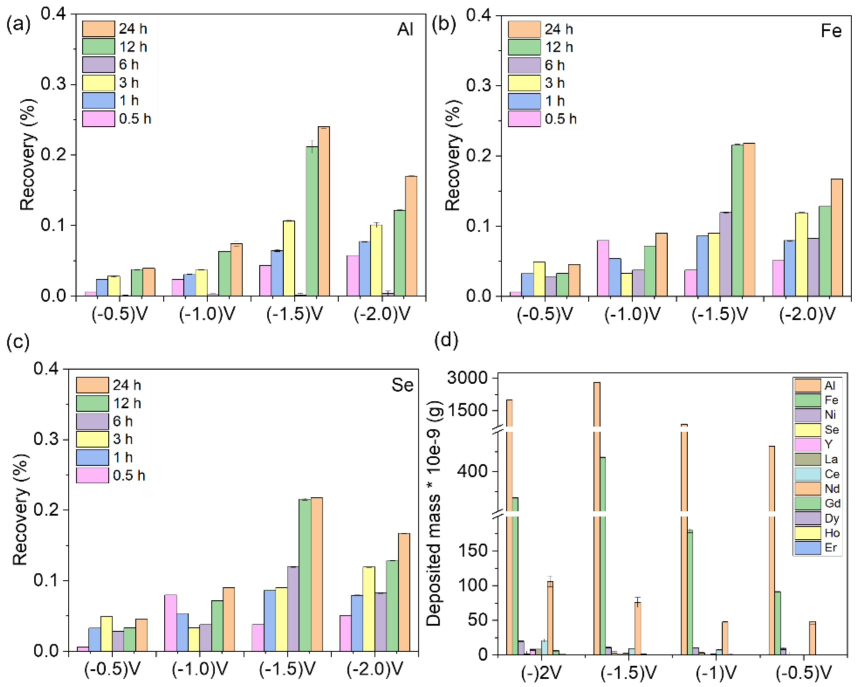

Figure S12: (a–c) Recovery (%) of co-deposited Al, Fe, and Se on electrodes from CFA-extracted REEs in [Hbet][Tf_2N], (d) Deposited mass of Al, Fe, and Se at −0.5, −1.0, −1.5, and −2.0 V vs Pt QRE across different deposition times. Error bars represent standard deviations from replicate measurements. QRE: quasi-reference electrode.

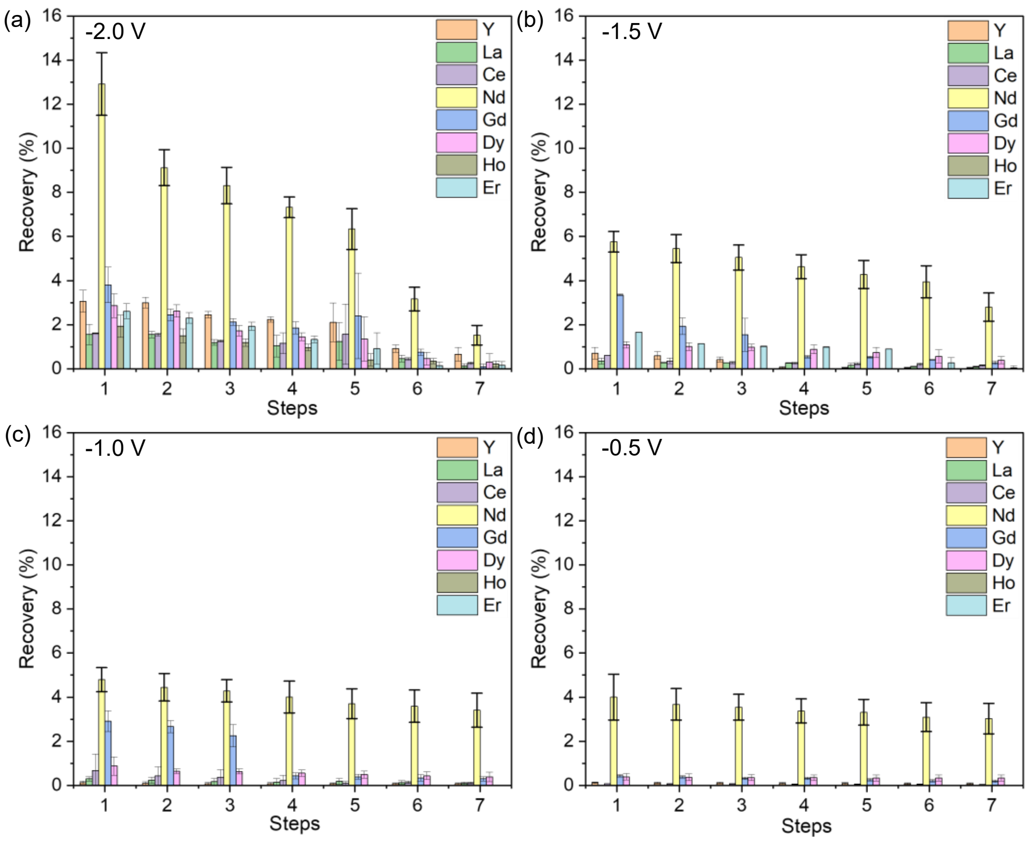

Fig S13: REEs recovery at -2.0 V to -0.5 V vs Pt QRE for 6 hours in multiple steps (a-d). QRE: Quasi-reference electrode.

Table S3: Separation factor for Nd from individual co-deposited REEs at -0.5 V vs Pt QRE to -2.0 V vs Pt QRE. QRE: Quasi-reference electrode.

	Separation factor (β)						
**Potential**	Y	La	Ce	Gd	Dy	Ho	Er
**(-0.5) V**	35.37	NA	57.36	11.64	9.80	NA	NA
**(-1.0) V**	48.45	22.63	14.06	3.04	7.06	NA	NA
**(-1.5) V**	16.83	21.34	15.34	3.72	5.46	NA	5.26
**(-2.0) V**	3.37	6.79	6.21	3.61	4.51	7.45	5.19

NA indicates that the element was either not detected by ICP or present at a concentration below the detection limit.

Table S4: Separation factor for Nd from overall co-deposited REEs at -0.5 V vs Pt QRE to -2.0 V vs Pt QRE. QRE: Quasi-reference electrode.

Separation factor ($\beta_{overall}$)

**Potential**	$\beta_{overall}$
**(-0.5) V**	36.78
**(-1.0) V**	11.0
**(-1.5) V**	10.75
**(-2.0) V**	5.05

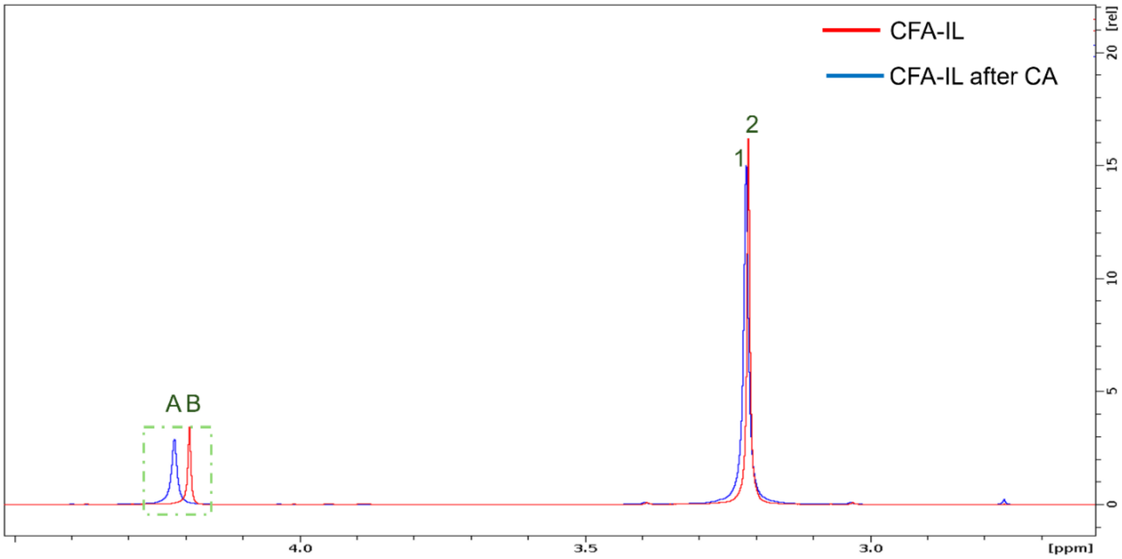

Figure S14: ^{1}H NMR spectra of CFA-extracted [Hbet][Tf_2N] before (red) and after electrodeposition at -2.0 V vs Pt QRE for seven steps at 6 h time interval (blue). Peaks A–B and 1–2 correspond to characteristic proton signals of the ionic liquid. QRE: Quasi-reference electrode.

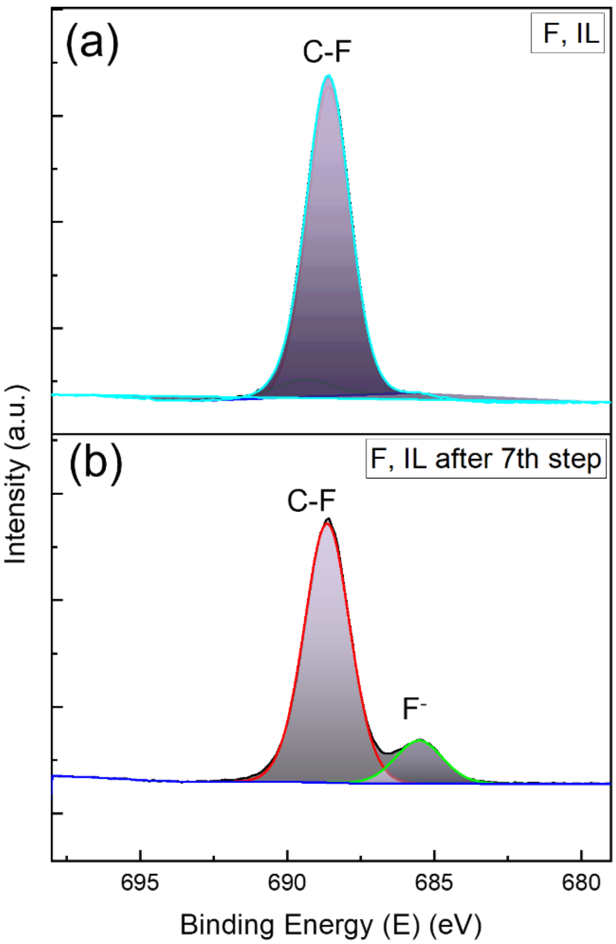

Figure S15: High-resolution XPS F 1s spectra of [Hbet][Tf_2N] before and after seven electrochemical cycles at -2.0 V vs Pt QRE for 6 h. QRE: Quasi-reference electrode.

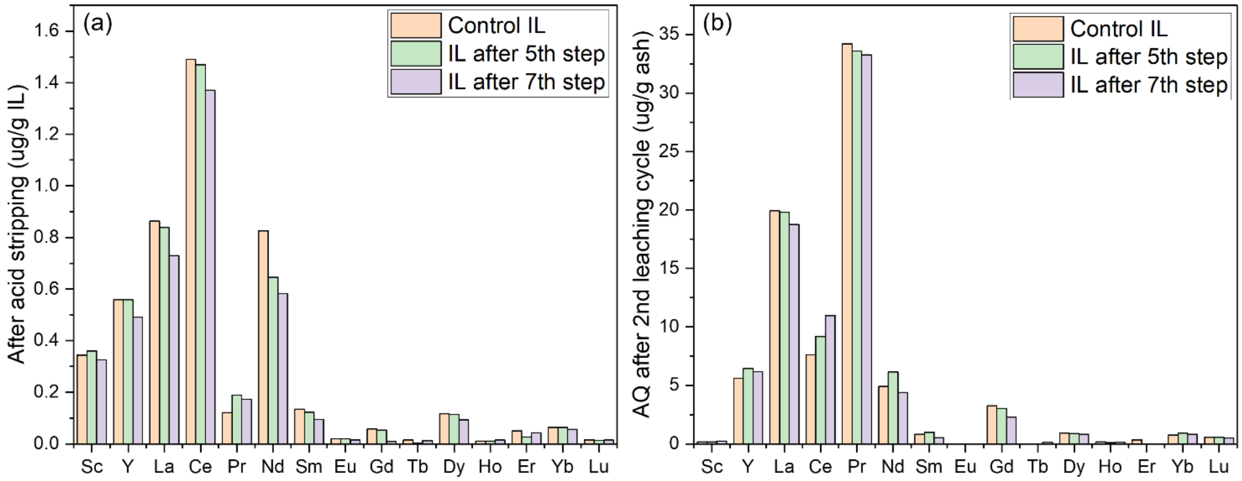

Fig S16: Regeneration of IL after 5 steps and 7 steps electrochemical recovery at 6 h at -2.0 V. The REE concentrations measured after acid stripping and in the aqueous phase following the second leaching cycle show comparable REE concentrations.

References

1. Taggart, R. K., Hower, J. C., Dwyer, G. S. & Hsu-Kim, H. Trends in the Rare Earth Element Content of U.S.-Based Coal Combustion Fly Ashes. *Environ Sci Technol* **50**, 5919–5926 (2016).

2. Pirpir, C., Yilmaz, O., Candirli, C. & Balaban, E. Evaluation of effectiveness of concentrated growth factor on osseointegration. *Int J Implant Dent* **3**, 7 (2017).

3. Kline, J. *Electrodeposition of Neodymium in the Room Temperature Ionic Liquid 1-Butyl-1-Methylpyrrolidinium Bis(Trifluoromethylsulfonyl)Imide and the Effects of Water and Ethanol: A Rotating Disk Electrode Study*. (University of Idaho, 2018).

4. Bourbos, E., Giannopoulou, I., Karantonis, A., Paspaliaris, I. & Panias, D. Electrodeposition of Rare Earth Metals from Ionic Liquids. in *Rare Earths Industry* 199–207 (Elsevier, 2016). doi:10.1016/B978-0-12-802328-0.00013-9.

5. Atifi, A., Baek, D. L. & Fox, R. V. Electrodeposition of Dysprosium in pyrrolidinium triflate ionic liquid at ambient temperature: Unraveling system efficiency and impact of solvation interplays on the reduction process. *Electrochim Acta* **378**, 138140 (2021).

6. *Standard Potentials in Aqueous Solutions*. (New-York - Basel, 1981).

7. *CRC Handbook of Chemistry and Physics*. (Taylow & Fracis Group, 2016).

8. Fihri, A., Artero, V., Pereira, A. & Fontecave, M. Efficient H2-producing photocatalytic systems based on cyclometalated iridium- and tricarbonylrhenium-diimine photosensitizers and cobaloxime catalysts. *Dalton Transactions* 5567 (2008) doi:10.1039/b812605b.
